# A Text Book of Ophthalmology and Ophthalmoscopy

**Published:** 1890-01

**Authors:** 


A TEXT BOOK OF OPHTHALMOLOGY AND OPHTHALMOSCOPY, by Dr. Hermann Schmidt–Rimpler, Professor of Opthalmology and director of the Ophthalmological Clinic in Marburg. Translated from the third German revised edition. Edited by D. B. St. John Roosa, M. D., LL. D., New York. William Wood & Co., Publishers. With 183 wood-cuts and three colored plates ; pp. 571.

The second edition of this splendid work was exhausted in less than two years and the third is now presented to the medical profession, after having been carefully revised and changes made as the knowledge of the subjects treated has been increased. The distinguished position of its authors, both in the old country and this, with their extensive facilities for investigating the subjects contained in this work, would commend it favorably to all those who are interested in this special department of the medical profession. It is printed with the best type and its general make up is all that could be desired in a book of its kind. Its general divisions are somewhat unlike similar books on the same subject, and its arrangement is admirably adapted to the wants of both the student of medicine and the busy practitioner. In fact, in looking through its pages we think we can safely say, that it is the equal at least of any recent work on the same subject. The authors have kept well up with the advances in this special department and no portion of the subject has not been well investigated. The book is divided into four general parts. The first of which treats of the methods of examining the eye, the errors of refraction and accommodation, amblyopia and amaurosis, taking each one in detail and explaining it so that it can be easily understood. The second part is devoted to Ohpthalmoscopy and the Ophthalmoscopic appearance in both healthy and diseased eyes, giving in detail the various changes that are readily seen with the Ophthalmoscope. Part third and fourth are devoted mainly to the pathological changes that take place in the lens, to diseases of the conjunctiva, cornea, the ocular muscles and lachrymal organs. The work as regards pathology and treatment of the various diseases connected with the eye is an exhaustive one. It is replete with both general and minor details, and would be a valuable addition to the library not only of the student of medicine, but to the distinguished specialist.

